# An isolated proximal tibiofibular joint dislocation in a young male playing soccer: a case report

**DOI:** 10.4076/1757-1626-2-7261

**Published:** 2009-07-27

**Authors:** Neil G Burke, Elaine Robinson, Neville W Thompson

**Affiliations:** Department of Orthopaedics and Trauma, Altnagelvin Area Hospital700 Glenshane Road, Londonderry, Northern Ireland, BT47 6SBUK

## Abstract

Isolated dislocation of the proximal tibiofibular joint is a rare injury. We present a 23-year-old caucasian man who sustained a traumatic anterolateral dislocation of the proximal tibiofibular joint. There is no consenus on definitive management, and we review the different published treatment and rehabilitation regimens for this injury. Our patient was successfully treated by open reduction and temporary Kirschner-wire fixation. The authors recommend their structured rehabilitation process involved using cast brace immobilization as allows for excellent soft tissue healing.

## Introduction

The proximal tibiofibular joint is a synovial joint located between the lateral tibial condyle and the fibular head. It has a fibrous joint capsule, which is reinforced by the anterosuperior and posterosuperior proximal tibiofibular ligaments. The posterolateral knee structures and the interosseous membrane provide additional stability. The proximal tibiofibular joint serves to dissipate lower leg torsional stresses and lateral tibial bending moments. It is also involved in transmitting axial loads during weight bearing [1].

Isolated proximal tibiofibular joint dislocation is an uncommon injury. Dislocation may result from direct or indirect trauma and is usually associated with sporting injuries or road traffic accidents [2-7]. Ogden [8] classified these injuries into four types (I, subluxation; II, anterolateral dislocation; III, posteromedial dislocation; IV, superior dislocation) with anterolateral dislocations accounting for approximately 85% of cases [2].

Anterolateral dislocations usually present with a prominent fibular head, localised tenderness and swelling. Ankle movement may exacerbate the knee pain. Anteroposterior (AP) and lateral x-rays are often diagnostic [9]; however, an axial computerised tomography scan may be performed to confirm the diagnosis and the direction of joint dislocation [10]. Closed reduction is usually successful; however, open reduction and temporary joint stabilisation may be required.

We report a case of isolated anterolateral dislocation of the proximal tibiofibular joint successfully treated by open reduction and temporary Kirschner-wire fixation. We discuss our choice of fixation and the post-reduction rehabilitation regimen.

## Case presentation

A 23-year-old Caucasian, male injured his right knee playing football when he collided with another player. He felt a ‘pop’ and immediately was unable to weight bear.

**Figure 1.**
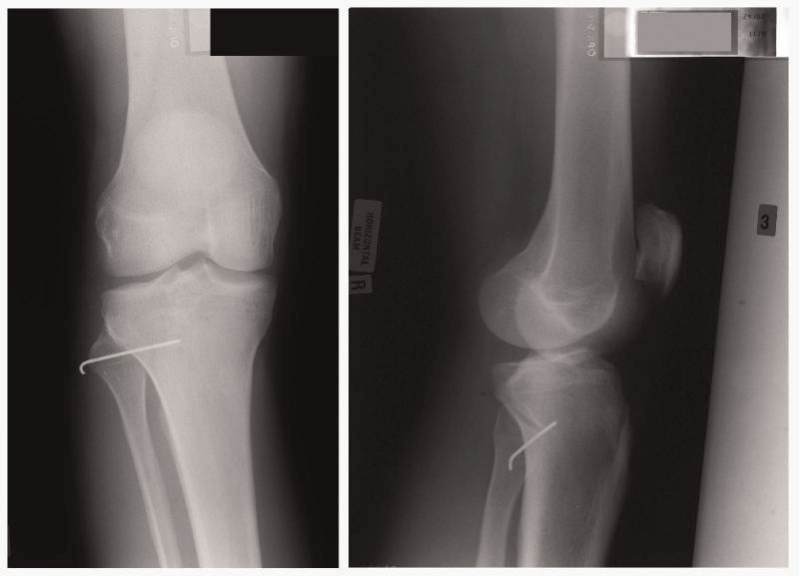
Anteroposterior and lateral views of the right knee demonstrating anterolateral dislocation of the fibular head.

On examination, a tender bony prominence was evident over the anterolateral aspect of the right knee. There was no effusion. Range of movement was reduced due to pain. There was no evidence of a peroneal nerve palsy. AP and lateral radiographs demonstrated an anterolateral (type II) dislocation of the proximal tibiofibular joint (Figure 1).

**Figure 2.**
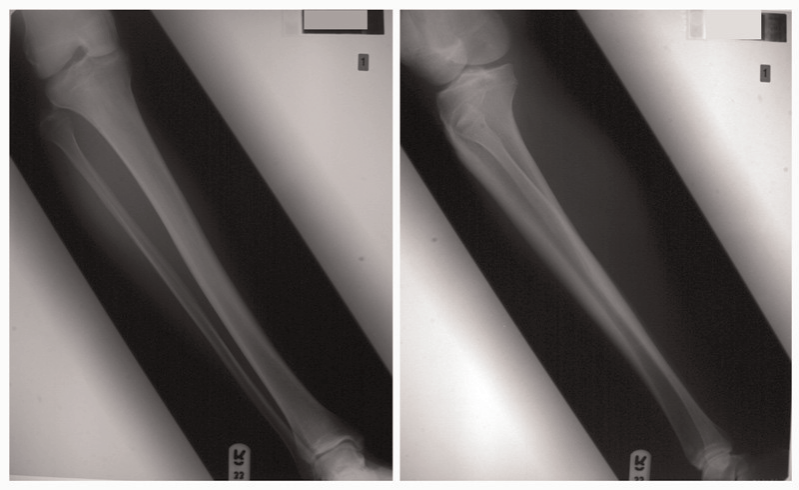
Anteroposterior and lateral views of the right knee demonstrating reduction of the fibular head and transfixation of the proximal tibiofibular joint with a single K-wire.

Attempts at closed reduction under general anaesthetic were unsuccessful. Open reduction was performed through a direct lateral approach taking care to protect the peroneal nerve. A bone lever was used to reduce the fibular head, which was grossly unstable following reduction. A single 1.6 mm trans-articular Kirschner-wire was inserted to stabilise the joint (Figure 2). The wire was cut and buried within the soft tissues. The anterior capsulo-ligamentous structures were repaired and the wound closed in layers. Postoperatively, a cast brace was applied with the hinges locked in 30° of knee flexion. He was mobilised non-weight bearing. At 4 weeks, the hinges were unlocked to allow unrestricted knee movement and full weight bearing. The cast brace and Kirschner-wire were removed 8 weeks post-injury to facilitate further rehabilitation. He was advised to refrain from sporting activities for 3 months.

Nine months from injury, the patient is asymptomatic with no clinical or radiological evidence of proximal tibiofibular joint instability. He has returned to his pre-injury level of sporting activities.

## Discussion

Isolated proximal tibiofibular joint dislocation is an uncommon injury with anterolateral dislocations accounting for the majority of cases [2].

Anterolateral dislocation results in injury to the anterior and posterior capsular ligaments and there may be an associated injury to the lateral collateral ligament (LCL) [9]. The main complication is injury to the peroneal nerve. Early diagnosis relies on a high index of suspicion as the symptoms may mimic a lateral meniscal injury or partial LCL disruption [4].

The initial management of an acute anterolateral dislocation is closed reduction (posterior pressure on the fibular head with the knee flexed 90° to relax the LCL and the foot externally rotated, everted and dorsiflexed to relax the interosseous membrane) [11]. Most dislocations can be reduced closed; however, open reduction may be necessary as anterior muscle tension and/or interposition of disrupted capsular ligaments may resist reduction. Acute dislocations undergoing open reduction require temporary joint stabilisation with either Kirschner wires [11] or screw fixation [6,12] combined with primary repair of the torn capsule and injured ligaments. Removal of the transfixing wire or screw is necessary to avoid fatigue fracture of the material. The use of bioabsorbable pins has been reported [5].

On reviewing the reported cases of anterolateral dislocation, there is no consensus as to the method or duration of immobilisation or the weight-bearing status following either closed or open reduction. Laing et al [4] reported a case of anterolateral dislocation treated by closed reduction, immediate mobilisation and application of a support bandage for 6 weeks. Ellis [3] reported a case of anterolateral dislocation treated by closed reduction without immobilisation. Falkenberg and Nygaard [7] reported a case of anterolateral dislocation treated by closed reduction and cast immobilisation for one week followed by 3 weeks in an elastic bandage with a progressive increase in weight bearing. Parkes and Zelko [11] reported a case of anterolateral dislocation treated by open reduction and temporary Kirschner-wiring. The patient was immobilised in a short leg cast non-weight bearing for 6 weeks. van den Bekerom et al [12] reported a case of anterolateral dislocation treated by open reduction and temporary screw fixation. They recommended full weight bearing but avoiding knee flexion beyond 90°. Robinson et al [6] recommended an above knee plaster for 6 weeks non-weight bearing whilst Rajkumar and Schmitgen [5] recommended a cylinder cast for 3 weeks partial weight bearing followed by a knee brace for 3 weeks. Removal of the temporary fixation in these cases was performed between 6 and 12 weeks from the index procedure [6,11,12].

In our case, closed reduction was unsuccessful, most likely due to tension in the anterior musculature as there was no soft tissue interposition. We opted to use a single, smooth Kirschner-wire for temporary fixation to minimise damage to the joint surfaces leaving it in-situ for 8 weeks to allow time for capsular and ligamentous healing. The purpose of initially immobilising the knee in a cast brace with the hinges locked in 30° of flexion was to de-tension the LCL and allow healing of any ligamentous disruption. Unlocking the hinges at 4 weeks but continuing with cast brace immobilisation allowed knee motion but prevented rotational stresses on the proximal tibiofibular joint. Full weight bearing was commenced at 4 weeks to promote mobility and muscle strength.

In conclusion, isolated dislocation of the proximal tibiofibular joint is an uncommon injury, which may require open reduction and temporary joint fixation. A structured post-reduction rehabilitation process is important. Furthermore, early diagnosis and treatment are important to prevent chronic knee pain and instability.

## Abbreviations

AP: anteroposterior
LCL: lateral collateral ligament
